# Rapamycin-Insensitive Up-Regulation of Adipocyte Phospholipase A2 in Tuberous Sclerosis and Lymphangioleiomyomatosis

**DOI:** 10.1371/journal.pone.0104809

**Published:** 2014-10-27

**Authors:** Chenggang Li, Erik Zhang, Yang Sun, Po-Shun Lee, Yongzhong Zhan, Yanan Guo, Juan C. Osorio, Ivan O. Rosas, Kai-Feng Xu, David J. Kwiatkowski, Jane J. Yu

**Affiliations:** 1 Brigham and Women’s Hospital/Harvard Medical School, Boston, Massachusetts, United States of America; 2 Peking Union Medical College, Beijing, China; Institute of Biosciences and Technology, Texas A&M Health Sciences Center, United States of America

## Abstract

Tuberous sclerosis syndrome (TSC) is an autosomal dominant tumor suppressor gene syndrome affecting multiple organs, including renal angiomyolipomas and pulmonary lymphangioleiomyomatosis (LAM). LAM is a female-predominant interstitial lung disease characterized by the progressive cyst formation and respiratory failure, which is also seen in sporadic patients without TSC. Mutations in *TSC1* or *TSC2* cause TSC, result in hyperactivation of mammalian target of rapamycin (mTOR), and are also seen in LAM cells in sporadic LAM. We recently reported that prostaglandin biosynthesis and cyclooxygenase-2 were deregulated in TSC and LAM. Phospholipase A2 (PLA2) is the rate-limiting enzyme that catalyzes the conversion of plasma membrane phospholipids into prostaglandins. In this study, we identified upregulation of adipocyte *AdPLA2 (PLA2G16)* in LAM nodule cells using publicly available expression data. We showed that the levels of AdPLA2 transcript and protein were higher in LAM lungs compared with control lungs. We then showed that TSC2 negatively regulates the expression of AdPLA2, and loss of TSC2 is associated with elevated production of prostaglandin E_2_ (PGE_2_) and prostacyclin (PGI_2_) in cell culture models. Mouse model studies also showed increased expression of AdPLA2 in xenograft tumors, estrogen-induced lung metastatic lesions of Tsc2 null leiomyoma-derived cells, and spontaneous renal cystadenomas from *Tsc2^+/−^* mice. Importantly, rapamycin treatment did not affect the expression of AdPLA2 and the production of PGE_2_ by TSC2-deficient mouse embryonic fibroblast (*Tsc2* ^−/−^MEFs), rat uterine leiomyoma-derived ELT3 cells, and LAM patient-associated renal angiomyolipoma-derived “mesenchymal” cells. Furthermore, methyl arachidonyl fluorophosphate (MAFP), a potent irreversible PLA2 inhibitor, selectively suppressed the growth and induced apoptosis of TSC2-deficient LAM patient-derived cells relative to TSC2-addback cells. Our findings suggest that AdPLA2 plays an important role in promoting tumorigenesis and disease progression by modulating the production of prostaglandins and may serve as a potential therapeutic target in TSC and LAM.

## Introduction

Tuberous sclerosis syndrome (TSC) is an autosomal dominant tumor suppressor gene syndrome characterized by neurologic disease, benign tumors in multiple organs, including renal angiomyolipomas, and pulmonary lymphangioleiomyomatosis (LAM), which is due to inactivating mutations in either *TSC1* or *TSC2* [1]. TSC1 and TSC2, together with TBC1D7, form a complex and act as a GTPase activating protein (GAP) to reduce Rheb-GTP levels [2], [3]. Loss of either TSC1 or TSC2 leads to increased Rheb activity which promotes mTORC1 activity and downstream phosphorylation of S6K, S6 and 4E-BP1, leading to increased protein synthesis for cellular growth and metabolism [4]. This understanding led to multiple preclinical studies which demonstrated the effectiveness of rapamycin, an mTORC1 inhibitor, in multiple animal models of TSC [5]–[11], which led to rapid clinical translation, and demonstration that rapamycin has clinical benefit for TSC kidney, lung, and brain tumors [12]–[15]. However, these tumors regrow when treatment is discontinued [12]–[15].

Although most biochemical and signaling effects in cells lacking TSC1 or TSC2 are thought to occur through activation of mTORC1, there is evidence that several abnormalities in TSC2-null cells are independent of mTORC1 [16]. For example, B-Raf kinase activity is reduced in TSC2-null cells due to high Rheb-GTP levels, but is independent of mTORC1 [17], [18]; and Tsc1 or Tsc2-null MEFs have a higher percentage of cilium-containing cells compared to controls, and rapamycin treatment has no effect on this observation [19]. Notch activation may also be regulated by TSC2 in an mTORC1-independent manner [5], although differing results have also been reported [20]. Recently we reported that TSC2 negatively regulates COX-2 expression, prostaglandin production and tumorigenesis in an mTORC1-insensitive, but mTORC2-dependent manner [10].

Prostaglandins play critical roles in chronic inflammation and cancer progression [21]. Prostaglandins are products of prostaglandin-endoperoxide synthases 1 and 2 (COX-1 and COX-2), which convert arachidonic acids released from phospholipase A2 (PLA2) -liberated membrane phospholipids, into prostaglandin G_2_ (PGG_2_)_,_ then PGH_2_. PGH_2_ is then converted into different prostacyclins and thromboxanes by specific isomerases [22], [23]. Increased prostaglandin production resulting from PLA2 upregulation may contribute to tumorigenesis via different mechanisms [23], [24].

Despite our previous findings that COX-2 expression and prostaglandin production were controlled by TSC2 [20], the relationship between prostaglandin biosynthesis and TSC/LAM pathogenesis has not been extensively studied. Here, we show upregulation of adipocyte-specific *AdPLA2* expression in LAM nodule cells relative to non-LAM lungs. We confirmed this finding by showing that the levels of AdPLA2 transcript and protein were higher in LAM lungs compared with control lungs. AdPLA2 accumulation is evident in LAM lung nodules and renal angiomyolipomas. Moreover, we found that TSC2 acts as a negative regulator of the expression of AdPLA2 and the production of prostaglandins in vitro and in vivo. Rapamycin treatment did not affect the expression of AdPLA2 and the production of PGE_2_ by TSC2-deficient cells. Finally, we show that pharmacologic inhibition of PLA2 selectively decreases the growth and promotes apoptosis of TSC2-deficient patient-derived cells relative to TSC2-addback cells.

## Materials and Methods

### Gene expression analysis

Re-analysis of previously published expression array data (GEO accession number GSE10072 [25], [26]; GSE19804 [25], [26] and GSE12027 [27]) were performed using the online tool GEO2R. Expression levels of PLA2 family members were compared among LAM cells collected by laser-capture microdissection from LAM nodules (LAM) and Non-LAM lungs (NL) including lung cancer/tumor.

### Ethics statement

The study protocol was reviewed and approved by the Partners Human Research Committee (PHRC) of the Brigham and Women’s Hospital and The Peking Union Medical College in China. After explanation of the description of the study, the risks and benefits of the participation, all participants signed a written consent form.

### Human samples

Lung tissue from LAM and control subjects was obtained from the National Disease Research Interchange (NDRI) and the Brigham and Women’s Hospital-Pulmonary Division Lung Tissue Biorepository. Informed consent was obtained for all lung tissues under Partners approved IRB protocols. Sera from 11 clinical LAM patients (10 sporadic LAM, one TSC-LAM) were collected from Peking Union Medical College in China, with informed consent obtained for research use.

### Quantitative RT-PCR

RNA from cultured cells and lung tissues was isolated using RNeasy Mini Kit (Qiagen). Gene expression was quantified using One-Step qRT-PCR Kits (Invitrogen) in the Applied Biosystems Real-Time PCR System and normalized to beta-actin.

### Cell culture and reagents

*Tsc2* ^−/−^ *p53* ^−/−^ and *Tsc2* ^+/+^ *p53* ^−/−^ mouse embryonic fibroblasts (MEFs) were developed from E10–12.5 embryos collected from *Tsc2* ^+/−^ or *Tsc2* ^+/−^ *p53* ^−/−^ intercrosses [28]. Cell culture media and supplements were from GIBCO (Frederick, MD). An immortalized TSC2-deficient human cell line derived from angiomyolipoma of a LAM patient [29], and its corresponding TSC2-rescued control cell line has been described previously [30]. Eker rat uterine leiomyoma-derived Tsc2-deficient cells (ELT3) were developed by Howe et al. [31], [32]. Cells were cultured in DMEM/F12 supplemented with 10% FBS, 0.2 µM hydrocortisone, 0.1 nM triiodothyronine, 0.01 µU/ml vasopressin, 1.6 µM FeSO4, cholesterol, ITS, 100 ng/ml EGF, 100 µg/ml zeomycin, and 1% penicillin-streptomycin-amphotericin B (PSA). Experiments were performed in 6–12 replicas for biochemical analyses. Cells were seeded at a density of 2.5×10^5^ cells/mL in 6-well plates in regular growth media for 24 hr, and then treated with inhibitors or vehicle in serum-free media for 24 hr. Cell-free conditioned media was collected, and cell lysates were prepared using RIPA (Boston Bioproducts, Boston, MA) or mPER lysis buffer (Pierce) supplemented with protease inhibitor cocktail (Roche, Indianapolis, IN) and phosphatase inhibitor cocktail (Thermo Scientific, Waltham, MA). Protein concentration was determined using the Bradford assay (BioRad Laboratories Inc. Hercules, CA).

### Pathway inhibitors

Methyl arachidonyl fluorophosphate (MAFP, 2–10 µM, Cayman Chemical), PD98059 (50 µM, Cell Signaling Technology), PI-103 (5 µM, Tocris), rapamycin (20 nM, Biomols), and Torin 1 (250 nM, Tocris) were used as indicated.

### Cell viability assay

Cells were seeded at a density of 5×10^4^/ml in 96-well plates for 24 hr, and then treated with inhibitors or vehicle for 24 hr. Cell viability was determined by MTT assay (Sigma). Values are expressed as mean ± SEM; n = 8/group. *P<0.05; Student’s t-test.

### Animal studies

The Brigham and Women’s Hospital-Children’s Hospital of Boston Standing Committee on Animals approved all procedures described according to standards as set forth in The Guide for the Care and Use of Laboratory Animals. *Tsc2* ^+/−^ mice (kindly provided by Dr. Kwiatkowski) develop renal cystadenomas at high frequency by 15 months of age [33]. In the current study, 15 months-old *Tsc2* ^+/−^ C57Bl6 mice were treated with celecoxib (0.1% in mouse chow) or vehicle (n = 3 mice) for one month, as described in [10]. Mice were sacrificed and kidneys were harvested. Xenograft tumor model (n = 8 mice/group) was established as previously described [11]. Animal health was monitored three days/week during the entire tumor experiments. The endpoint of the xenograft tumor study was the onset of the clinical signs of pain/distress including 1) animals are in constant pain (hunched posture, sluggish movement, vocalization when handled); 2) bilateral tumors (two subcutaneous tumors/mouse) have caused inactivity, became ulcerated and/or larger than 15% of the animal’s body weight (tumor volume ∼1,000 mm^3^); 3) animals have lost more than 20% of their body weight. All mice were euthanized by carbon dioxide (CO_2_) inhalation via compressed gas in response to the onset of the above distress.

### Immunohistochemistry

Immunohistochemistry was performed on paraffin-embedded 10 µm-sections using antibodies against AdPLA2. Slides were deparaffinized and antigen retrieval was performed using sodium citrate retrieval solution pH 6. Sections were stained by the immunoperoxidase technique using DAB substrate (Invitrogen) and counterstaining with hematoxylin.

### Immunoblotting analysis

Protein samples were analyzed by SDS-PAGE using 4–12% NuPAGE Gel (Invitrogen), and transferred to a nitrocellulose membrane. Immunoblotting was performed by standard methods using HRP-conjugated secondary antibodies, and chemiluminescence using Supersignal West Pico Chemiluminescent substrate (Thermo Scientific). Antibodies used: AdPLA2, Phospho-Erk1/2 (T202/Y204), Phospho-S6 (S235/236), cleaved caspase 3, cleaved PARP (Cell Signaling Technology); tuberin (Santa Cruz); smooth muscle actin (BioGenex); and beta-actin (Sigma).

### Quantification of prostaglandin metabolites and VEGF-D

PGE_2_ and 6-keto-PGF_1α_ were measured using Enzyme Linked Immuno-Sorbent Assay (ELISA) kits (Cayman Chemical). Levels of secreted prostaglandins were normalized to protein concentrations and expressed as pg/mg protein. Serum levels of PGE_2_ and VEGF-D were quantified using ELISA kits.

### Statistical analyses

Statistical analyses were performed using Student’s *t*-test when comparing two groups. Results are presented as means ± SEM.

## Results

### Identification of adipocyte-specific AdPLA2 upregulation in pulmonary LAM nodule cells

**Figure 1:**
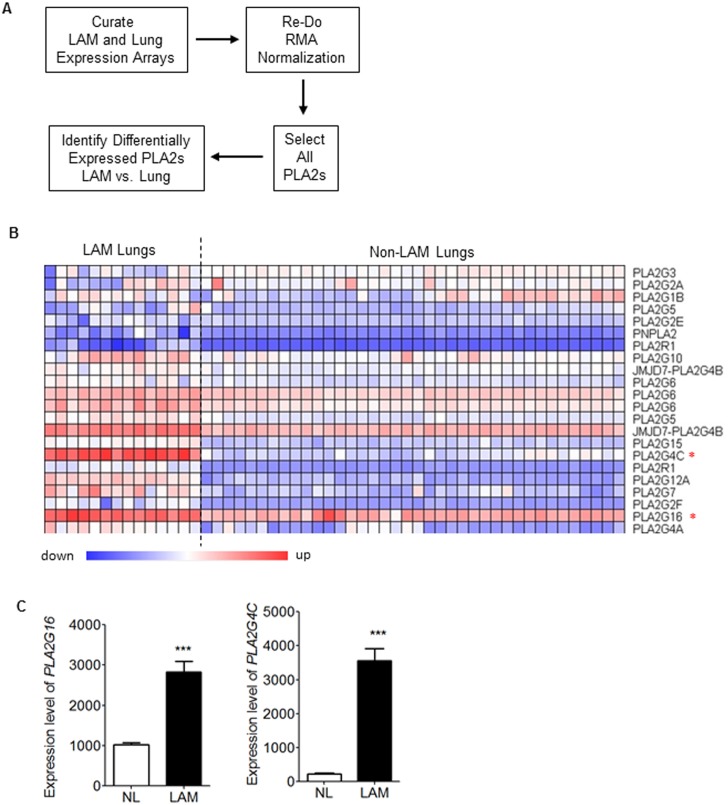
Identification of adipocyte AdPLA2 in LAM associated angiomyolipoma-derived cells and pulmonary LAM nodules using public expression arrays. (A) Workflow of the expression array analysis of PLA2s using public available gene expression arrays. (B) A heatmap of the expression of all PLA2s from the re-analysis of previously published expression array data [25], [27],[34]. (C) The transcript levels of *PLA2G16* (phospholipase A2, group XVI, AdPLA2) and *PLA24C* (phospholipase A2, group IVC, cytosolic, calcium-independent) in LAM lungs (LAM, n = 14) and Non-LAM lungs (NL, n = 49). ***P<0.005, Student’s t-test.

Our previous studies had identified an estradiol-enhanced prostaglandin biosynthesis signature in Tsc2-deficient (TSC2−) cells [20]. Since prostaglandin biosynthesis is initiated by PLA2 acting on membrane phospholipids, we examined its expression using public available expression array data sets from pulmonary LAM nodule cells collected by laser-capture microdissection (GEO data set GSE12027) [27], lung cancer/tumor (GEO data set GSE10072 and GSE19804) [25], [34], and control lungs (GEO data set GSE10072 and GSE19804) [25], [34] (**Figure 1A**). The transcript levels of 18 PLA2 family members in LAM nodule cells were compared to non-LAM lungs (NL), respectively (**Figure 1B**). Of note, two PLA2 transcripts, *PLA2G16* (AdPLA2) and *PLA2G4C*, were significantly higher by two-fold in LAM nodule cells compared to control non-LAM lungs (p<0.05, **Figure 1C**).

### Expression of AdPLA2 is upregulated in pulmonary LAM nodules and angiomyolipomas

**Figure 2:**
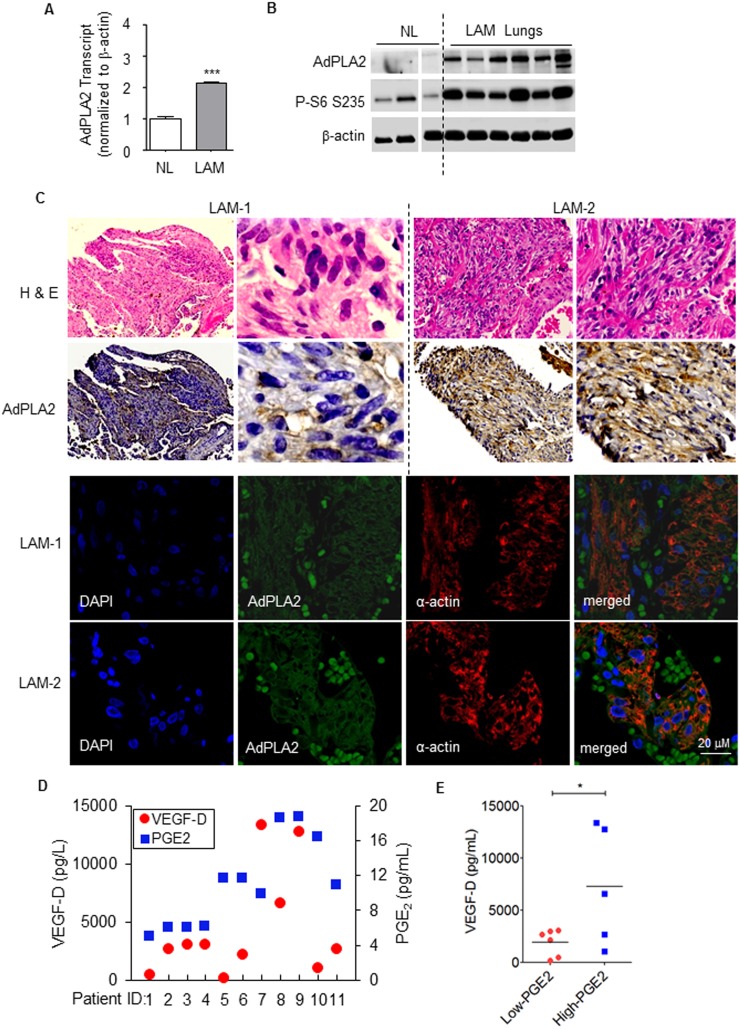
Expression of AdPLA2 is upregulated in pulmonary LAM. (A) Real-time RT-PCR analysis of the transcript levels of *AdPLA2* in LAM lungs (LAM) relative to normal lungs (NL). Data show the mean of five sets of independent samples. (B) Immunoblotting analysis of AdPLA2 and phospho-S6 in lung lysates from pulmonary LAM subjects (LAM lungs, n = 6) and from normal lungs (NL, n = 3). (C) Immunohistochemical and immunofluorescent staining of smooth muscle actin (α-actin), phospho-S6 (P-S6) (S235/236) and AdPLA2 in pulmonary LAM nodules from two LAM subjects (LAM-1 and LAM-2). (D) Serum levels of VEGF-D and PGE_2_ were measured using ELISA from 11 clinical LAM patients. (E) The correlation of serum levels of PGE_2_ and VEGF-D was analyzed. *P<0.05, ***P<0.005, Student’s t-test.

To validate the expression array data, the transcript levels of *AdPLA2* were examined in six LAM lungs and six control lungs using real-time RT-PCR. LAM lungs (LAM) exhibited a twofold increase of AdPLA2 transcript relative to control lungs (NL) (p<0.01, **Figure 2A**), confirming the overexpression of AdPLA2 identified using LAM expression array analyses (Figure 1). Moreover, immunoblotting analysis showed that LAM lungs positive for Phospho-S6 (S235/236) accumulated higher levels of AdPLA2 protein relative to control normal lungs (NL) (**Figure 2B**). Furthermore, immunohistochemical staining of two LAM specimens showed abundant accumulation of AdPLA2 in smooth muscle-like cells (**Figure 2C**). In addition, confocal microscopy showed that smooth muscle actin-positive cells were stained with AdPLA2, whereas pulmonary artery cells were negative with AdPLA2 staining (**Figure 2C**).

Serum levels of VEGF-D have been used a biomarker for LAM [35], [36]. We recently reported that serum levels of PGE_2_ were higher in women with LAM relative to healthy women [20]. To determine the correlation of the levels of VEGF-D and PGE_2_ in LAM, we collected sera from 11 LAM patients (**Table 1**) and measured the levels of PGE_2_ and VEGF-D using ELISA. Serum levels of PGE_2_ segregated into two groups: 5–10 pg/mL (high-PGE_2_) and 10–20 pg/mL (low PGE_2_, **Figure 2D**). Interestingly, serum levels of VEGF-D were lower in LAM subjects (VEGF-D 1937±520 pg/mL) with lesser PGE_2_ compared to LAM subjects (VEGF-D 7298±2529 pg/mL) with higher PGE_2_, indicative of a strong correlation between PGE_2_ and VEGF-D (p<0.05, **Figure 2E**). These data suggest that serum PGE_2_ could be useful as a diagnostic marker and for assessment of disease severity.

**Table 1:** Clinical profile of LAM subjects.

ID	Age	Type of LAM(sporadic or TSC)	FEV1 (%)	FVC	FEV1/FVC	6MWT	PaO_2_	PA-aO_2_	Pneumo-thorax	Pleuraleffusion/chylothotax
1	38	Sporadic	n.a.	n.a.	n.a.	n.a.	n.a.	n.a.	No	chylothotax
2	46	Sporadic	77.0	126.8	51.8	463	33.1	36.4	No	No
3	48	Sporadic	34.1	87.2	33.8	433	76.6	33.4	No	No
4	29	Sporadic	n.a.	n.a.	n.a.	n.a.	n.a.	n.a.	Yes	chylothotax
5	48	Sporadic	n.a.	n.a.	n.a.	510	85.2	27.3	Yes	No
6	27	Sporadic	84.5	86.5	85.2	520	73.5	40.4	n.a.	n.a.
7	45	Sporadic	64.1	99.8	55.2	n.a.	93.6	21.1	No	chylothotax
8	46	Sporadic	86.7	96.8	76.4	360	80.2	26.2	No	No
9	40	TSC	84.8	82.6	88.2	505	106	n.a.	No	No
10	48	Sporadic	n.a.	n.a.	n.a.	337	81.7	25.2	Yes	chylothotax
11	47	Sporadic	n.a.	n.a.	n.a.	224	56.7	53.9	N o	chylothotax

Definition of abbreviations: ID = patient number; LAM = Lymphangioleiomyomatosis; TSC = Tuberous sclerosis; FEV1 (%) = Forced Expiratory Volume in 1 second (% predicted); FVC = Forced vital capacity; 6MWT = 6-minute-walk-test; PaO_2_ = partial pressure of oxygen in arterial blood; PA-aO_2_ = Alveolar-arterial gradient; n.a. = not available.

### TSC2 negatively regulates AdPLA2 expression in a rapamycin-insensitive manner in vitro

**Figure 3:**
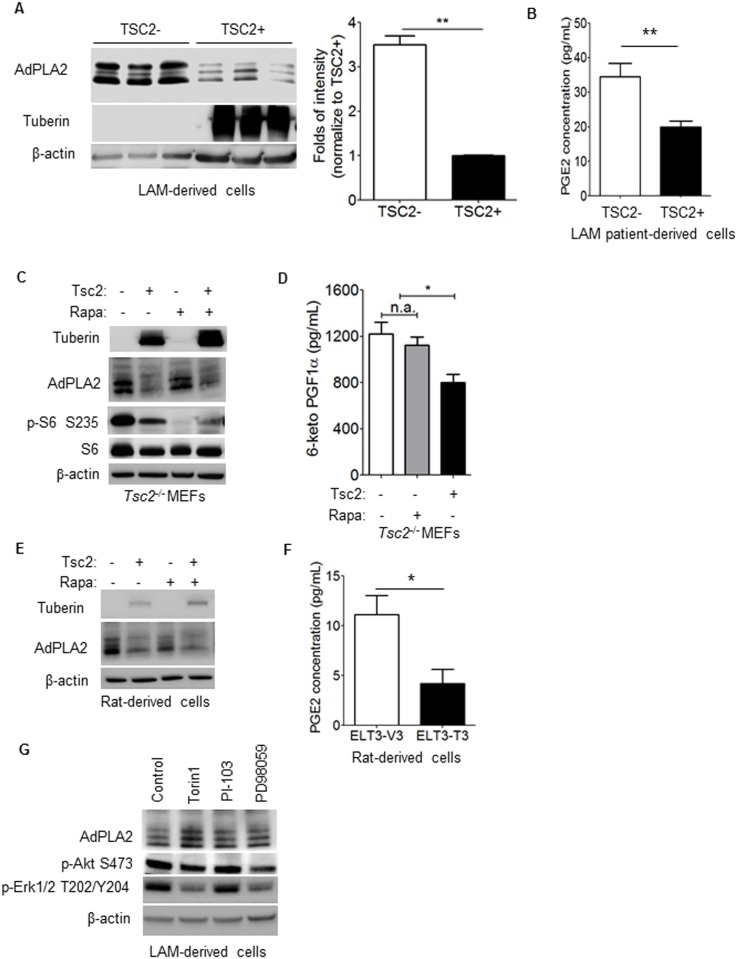
TSC2 negatively regulates AdPLA2 expression in rapamycin-insensitive manner in vitro. (A) Immunoblotting analysis of AdPLA2 and tuberin in TSC2-deficient (TSC2−) and TSCS2-addback (TSC2+) LAM patient-derived cells. Data show the mean of three sets of independent samples. Densitometry analysis of the protein levels of AdPLA2. (B) Secreted levels of prostaglandin E_2_ (PGE_2_) were quantified in conditioned media collected from TSC2-deficient (TSC2−) and TSC2-addback (TSC2+) LAM patient-derived cells using ELISA. Results are representative of three sets of independent samples per group. (C) *Tsc2* ^−/−^ *p53* ^−/−^ and *Tsc2* ^+/+^ *p53* ^−/−^MEFs were treated with 20 nM rapamycin for 24 hr. Levels of AdPLA2, tuberin and phospho-S6 (S235/236) were assessed by immunoblotting analysis. Results are representative of three different experiments. (D) *Tsc2* ^−/−^ *p53* ^−/−^ and *Tsc2* ^+/+^ *p53* ^−/−^ MEFs were treated with 20 nM rapamycin (Rapa) or control for 24 hr. Secreted levels of 6-keto-PGF_1α_ were quantified in conditioned media using ELISA. Results are representative of three sets of independent samples per group. (E) Rat-derived ELT3 cells were treated with 20 nM rapamycin (Rapa) or control for 24 hr. Immunoblotting analysis of AdPLA2 and tuberin were assessed. Results are representative of three different experiments. (F) Secreted levels of prostaglandin E_2_ (PGE_2_) were quantified in conditioned media collected from TSC2-deficient (TSC2−) and TSC2-addback (TSC2+) ELT3 cells using ELISA. Results are representative of three sets of independent samples per group. (G) Patient-derived TSC2-deficient (TSC2−) cells were treated with 20 nM rapamycin (Rapa), 100 nM Torin1, 50 µM PI-103, 50 µM PD98059 or control for 24 hr. Levels of AdPLA2, tuberin, phospho-Akt (S473) and phospho-Erk (T202/Y204) were assessed by immunoblotting analysis. *P<0.05, **P<0.01, Student’s t-test.

To define the molecular mechanisms responsible for AdPLA2 upregulation in LAM, we first tested whether TSC2 plays a role in regulating AdPLA2 expression. AdPLA2 expression was higher by 3.5-fold in TSC2-deficient LAM patient-derived cells (TSC2−) compared with TSC2-addback cells (TSC2+) (p<0.001, **Figure 3A**). Because PLA2 catalyzes the conversion of plasma membrane phospholipids to prostaglandins, we next measured the production of PGE_2_ in cells lacking TSC2. TSC2-deficient LAM patient-derived cells secreted ∼75% higher levels of PGE_2_ compared with TSC2-addback cells (p<0.01, **Figure 3B**), indicative of active AdPLA2.

To determine whether mTORC1 mediates AdPLA2 upregulation, rapamycin treatment was employed in *Tsc2* ^−/−^ *p53* ^−/−^ and *Tsc2* ^+/+^ *p53* ^−/−^ MEFs [33], Tsc2-deficient [32], [37] and TSC2-addback [38] rat uterine-leiomyoma-derived ELT3 cells, and TSC2-deficient LAM patient-derived cells [29]. Rapamycin treatment drastically reduced phosphorylation of S6, but had no effect on AdPLA2 or S6 expression in *Tsc2* ^−/−^ *p53* ^−/−^ MEFs (**Figure 3C**). We also found that the secreted levels of 6-keto PGF_1α_, a prostaglandin metabolite, were elevated by 50% in *Tsc2* ^−/−^ *p53* ^−/−^ MEFs relative to *Tsc2* ^+/+^ *p53* ^−/−^ MEFs (p<0.05, **Figure 3D**). Moreover, rapamycin treatment did not affect the levels of 6-keto PGF_1α_ (**Figure 3D**). Furthermore, in rat-derived cells, the protein levels of AdPLA2 were higher in Tsc2-deficient ELT3 cells (Tsc2−) compared with TSC2-reexpressing cells (TSC2+) (**Figure 3E**). Rapamycin treatment also did not alter AdPAL2 expression (**Figure 3E**). Similarly, secreted levels of PGE_2_ were also elevated in by ∼67% in Tsc2-deficient cells (ELT3-V3, Tsc2−) relative to TSC2-reexpressing cells (ELT3-T3, TSC2+) (p<0.05, **Figure 3F**).

Since rapamycin is an allosteric partial inhibitor of mTORC1 only [39], we also examined the effects of Torin1, a potent ATP-competitive mTORC1 and mTORC2 inhibitor [40] on AdPLA2 expression. Torin 1 reduced levels of P-Erk1/2 (T202/Y204) and P-Akt (S473), but did not affect AdPLA2 expression in LAM patient-derived cells (**Figure 3G**). Furthermore, neither Akt inhibition (PI-103) nor MEK1/2 inhibition (PD98059) affected AdPLA2 expression (**Figure 3G**). Together, our data suggest that upregulation of AdPLA2 expression is independent of mTOR, Akt and MEK1/2 signaling pathways in cells lacking TSC2.

### TSC2 negatively regulates AdPLA2 expression in vivo

**Figure 4:**
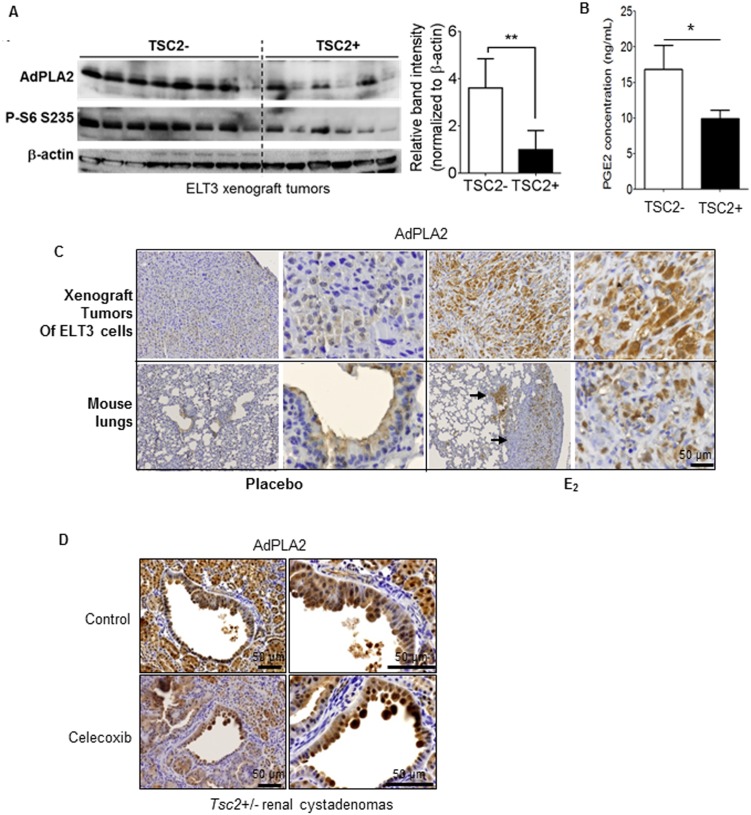
TSC2 negatively regulates AdPLA2 expression in vivo. Female CB17-scid mice were subcutaneously inoculated with ELT3-V3 cells (TSC2-, vector) or ELT3-T3 (TSC2+, TSC2 addback) cells (n = 8 mice/group). (A) Immunoblotting analysis of AdPLA2 and phospho-S6 (S235/236) in xenograft tumors of ELT3 cells. A densitometry analysis of AdPLA2 was performed. (B) Urinary levels of PGE_2_ were quantified using ELISA and normalized to creatinine levels in mice bearing xenograft tumors. Results are representative of five to nine mice per group. C) Immunohistochemical staining of AdPLA2 in xenograft tumors of Tsc2-deficient rat-derived ELT3 cells and lungs of mice treated with placebo or estrogen. Arrowheads point to lung metastatic lesions in estradiol-treated mice bearing xenograft tumors of ELT3 cells. (D) *Tsc2^+/−^* mice were treated with either vehicle or Celecoxib (Pfizer) (0.1% in mouse chow) for one month, and then sacrificed for analysis at the end of treatment (n = 3 mice/group). Immunohistochemical staining of AdPLA2 in renal cystadenomas from *Tsc2* ^+/−^mice was performed. *P<0.05, **P<0.01, Student’s t-test.

To determine whether TSC2 regulates AdPLA2 expression in vivo, we first used xenograft tumors from mice inoculated with TSC2-deficient ELT3-V3 (TSC2−) cells and TSC2-reexpressing ELT3-T3 (TSC2+) cells. AdPLA2 protein levels were markedly higher by 3.5-fold in TSC2- xenograft tumors with increased phospho-S6 compared to that of TSC2+ tumors (**Figure 4A**). Mice bearing xenograft tumors of ELT3-V3 (TSC2−) produced higher urinary levels of PGE_2_ compared with mice bearing ELT3-T3 (TSC2+) xenograft tumors (p<0.05, **Figure 4B**). The fact that LAM is a female predominant disease made us hypothesize that estradiol plays an important role in disease progression. To assess the impact of estradiol on AdPLA2 expression in vivo, ovariectomized female scid mice inoculated with Tsc2-deficient ELT3 cells were treated with estradiol or placebo. Xenograft tumors were developed, as expected. The accumulation of AdPLA2 in xenograft tumor was markedly elevated in mice receiving estradiol compared with placebo treatment (**Figure 4C**). Importantly, AdPLA2 accumulation was evident in lung metastatic lesions of ELT3 cells in mice treated with estradiol compared with placebo treatment (**Figure 4C**). These data suggest that estradiol-enhanced AdPLA2 expression was correlated with xenograft tumor growth and lung metastasis of Tsc2-deficient cells.

We next examined spontaneously arising renal cystadenomas in *Tsc2* ^+/−^ mice [33]. Renal Tumors exhibited increased expression of AdPLA2 relative to stromal cells, although some normal kidney tubule cells were moderately positive for AdPLA2, while glomeruli were negative for AdPLA2 (**Figure 4D**). We recently reported that TSC2 negative regulates cyclooxygenase-2 (COX-2) and prostaglandin biosynthesis [10]. To examine the impact of COX-2 inhibition on the upstream regulator, AdPLA2, *Tsc2* ^+/−^ mice were subjected to short-term treatment with COX-2 specific inhibitor-Celecoxib or vehicle control before harvesting renal tumors for immunoblotting. The levels of AdPLA2 expression from a *Tsc2* ^+/−^ mouse treated with Celecoxib remained high in renal tumors compared to adjacent normal kidney tissues (**Figure 4D**), suggesting that COX-2 targeted therapy does not have a feedback effect on AdPLA2 expression.

### Pharmacological inhibition of PLA2 selectively suppresses the growth of TSC2-deficient LAM patient-derived cells

**Figure 5:**
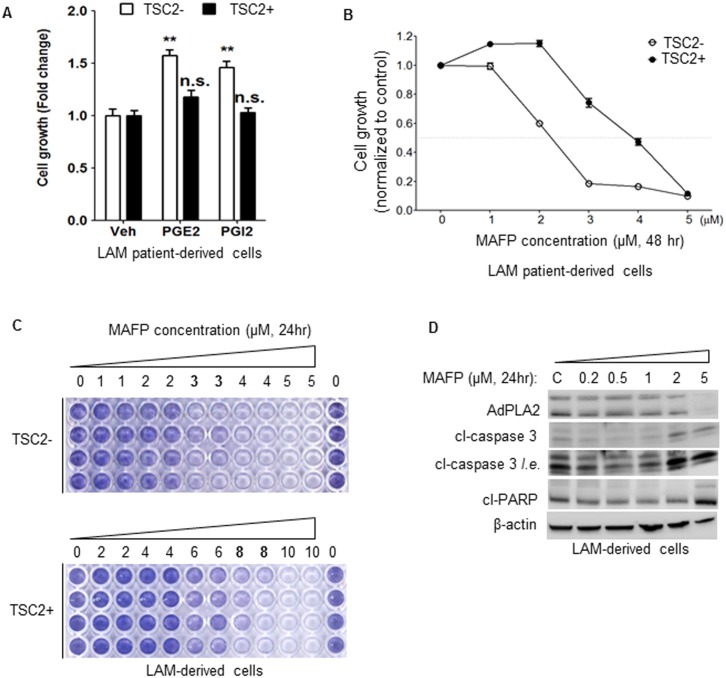
Inhibition of AdPLA2 selectively suppresses the growth of LAM patient-derived cells. (A) TSC2-deficient (TSC2−) and TSCS2-addback (TSC2+) LAM patient-derived cells were treated with 100 nM PGE_2_, 100 nM PGI_2_, or vehicle control for 72 hr. Cell proliferation was measured using MTT assay. Results are representative of the average of twelve sets of independent samples per group. (B) TSC2-deficient (TSC2−) and TSC2-addback (TSC2+) LAM patient-derived cells were treated with PLA2 inhibitor methyl arachidonyl fluorophosphonate (MAFP) for 48 hr. Cell growth was measured using MTT assay. Results are average of twelve sets of independent samples per group. (C) TSC2-deficient (TSC2−) and TSCS2-addback (TSC2+) LAM patient-derived cells were treated with PLA2 inhibitor MAFP for 24 hr. Cell proliferation was measured using crystal violet staining. Results are representative of three sets of independent experiments. (D) LAM patient-derived TSC2-deficient (TSC2−) cells were treated with MAFP at various concentrations for 24 hr. Levels of AdPLA2, cleaved-caspase 3 and cleaved-PARP was assessed by immunoblotting analysis. Results are representative of three different experiments. **P<0.01, Student’s t-test.

To determine whether elevated PGE_2_ production has any biologic consequence, we examined the cell growth in response to PGE_2_ and PGI_2_ stimulation. PGE_2_ or PGI_2_ stimulation for 72 hr led to a 60% or 55% increase of the growth of TSC2-deficient cells compared with vehicle control, respectively (p<0.01, **Figure 5A**), although the growth of TSC2-addback cells was not sensitive to PGE_2_ or PGI_2_ stimulation (**Figure 5A**). Because mTORC1 inhibition had no effect on AdPLA2 expression, we examined whether the PLA2 inhibitor, methyl arachidonyl fluorophosphonate (MAFP), a selective, active-site directed, irreversible inhibitor of PLA2 [41], had an effect on the growth of TSC2-deficient cells. Treatment with 3 µM MAFP significantly reduced the number of TSC2-deficient cells compared to vehicle control (p<0.05, **Figure 5B**). Importantly, TSC2-addback cells were resistant to 3 µM MAFP treatment, although 4.5 µM MAFP reduced cell numbers by 80% relative to vehicle control (**Figure 5B**). To further define the optimal dose of MAFP in promoting cell death, we treated TSC2-deficient cells with 1, 2, 3, 4, and 5 µM MAFP, and TSC2-reexpressing cells with 2, 4, 6, 8, and 10 µM MAFP. 3 µM MAFP markedly reduced cell variability in TSC2-deficient LAM patient-derived cells (p<0.05, **Figure 5C**). In contrast, 8 µM MAFP decreased the variability of TSC2-addback cells compared with vehicle control (**Figure 5C**). Our data indicate that MAFP selectively suppresses the growth of TSC2-deficient cells relative to TSC2-addback cells. Moreover, 2 µM MAFP treatments led to an increased caspase 3 cleavage, and 5 µM MAFP caused higher levels of cleaved caspase 3 and PARP compared with control treatment (**Figure 5D**). Together, these data indicate that inhibition of PLA2 selectively suppresses the survival of TSC2-deficient LAM patient-derived cells.

## Discussion

Recently we reported that COX-2 and prostaglandin biosynthesis pathway is aberrantly activated in TSC2-deficient cells [10]. PGE_2_ and PGI_2_, in particular, two major prostaglandin metabolites of the eicosanoid end-products from the COX-2 mediated branch of the arachidonate pathway, were significantly elevated in TSC2-deficient cells, in preclinical models of TSC/LAM, and in sera from women with LAM [10]. Importantly, pharmacological inhibition of COX-2 led to apoptosis of TSC2-deficient cells, and blocked the progression of Tsc2-deficient xenograft tumors and kidney cystadenomas in preclinical models [10]. The findings of elevated COX-2 activity and prostaglandin production in our recently published study [10] led us hypothesize that phospholipase A2 (PLA2), the first rate-limiting enzyme responsible for the conversion of plasma membrane phospholipids to arachidonate [42], is activated in cells lacking TSC2 and/or with mTORC1 hyperactivation.

Phospholipase A2s (PLA2s) belongs to a superfamily of 15 distinct members comprising four major clusters, including the secreted sPLA2, cytosolic cPLA2, calcium-independent iPLA2, and platelet activating factor (PAF) acetyl hydrolase/oxidized lipid lipoprotein associated (LP) PLA2 [43]–[45]. Each type of PLA2 plays a unique role in lipid metabolism and disease progression. To determine which PLA2(s) plays a critical role in TSC/LAM pathogenesis, we analyzed the publicly available expression array data sets from laser capture microdissected LAM nodule cells [27] and non-LAM lung data sets [25], [26]. Surprisingly, we found that the adipocyte-specific PLA2 (AdPLA2, also called PLA2G16) was upregulated in LAM nodule cells relative to non-LAM lungs (Figure 1). AdPLA2 is a major PLA2 enzyme in adipose tissue and regulates lipolysis through PGE_2_ [46], [47]. This finding is of particular interest because of the “mesenchymal” features of LAM, including the expression of smooth muscle actin [48] and melanocytic markers HMB45 [49] and/or gp100 [50], the metastatic potential [51], and epithelial-mesenchymal transition [26]. Pathologically, renal angiomyolipomas are composed of immature smooth muscle cells, aberrant blood vessels, and fat cells [52]. Fat cells are also evident in a primary cultures derived from a LAM-associated angiomyolipoma [29]. Thus, the identification of AdPLA2 is in agreement with the characteristics of LAM patient-derived cells.

Our current study demonstrated that the expression of AdPLA2 is negatively regulated by TSC2. However, the elevated AdPAL2 expression was not affected by pathway blockade, including mTORC1 inhibitor rapamycin, mTORC1/2 inhibitor Torin 1, Akt inhibitor PI-103, or MEK1/2 inhibitor PD98059, which represents uncovered regulatory mechanisms. Several precedent studies have documented possible mTORC1-independent cellular outcomes in cells lacking TSC1 or TSC2 (reviewed in Neuman et al. [53]). B-Raf kinase activity is reduced in TSC2-deficient cells due to Rheb-GTP, but independent of mTORC1 [17], [18]. Akt activation in *Tsc2* ^−/−^ *p53* ^−/−^ MEFs was reduced due to impaired mTORC2 activity [54]. *Tsc1* ^−/−^ or *Tsc2* ^−/−^ *p53* ^−/−^ MEFs had more abundant cilia relative to the counterpart controls, and rapamycin treatment had no effect on the cilia formation [19]. We had previously found that the overexpression of matrix metalloproteinase (MMP) in TSC2-deficient LAM patient-derived cells is insensitive to rapamycin [30]. Recently, we discovered that TSC2 negatively regulates COX-2 expression and prostaglandin production in a rapamycin-insensitive but mTORC2-dependent manner [10]. The current study adds to the accumulating evidence that mTORC1-independent regulation of signaling pathways may contribute to the pathogenesis and progression of TSC/LAM.

The Multicenter International LAM Efficacy of Sirolimus Trial (The MILES trial) demonstrated that the mTORC1 inhibitor Sirolimus stabilizes lung function and improves quality of life in LAM patients. However, upon drug discontinuation, lung function decline resumed [15], indicating that mTORC1 inhibitor has a cytostatic but not cytotoxic effect on LAM cells. There is an unmet need for novel strategies to promote cell death in TSC/LAM. In summary, we report that AdPLA2 expression is elevated in TSC2-deficient patient-derived cells compared to TSC2-addback cells, and that the PLA2 inhibitor selectively suppresses the proliferation of TSC2-deficient cells. We anticipate that PLA2 inhibitors may provide a novel therapeutic strategy for TSC and LAM.

## References

[1] Crino PB, Nathanson KL, Henske EP (2006) The tuberous sclerosis complex. N Engl J Med 355: 1345–1356. 17005952 10.1056/NEJMra055323

[2] Plank TL, Yeung RS, Henske EP (1998) Hamartin, the product of the tuberous sclerosis 1 (TSC1) gene, interacts with tuberin and appears to be localized to cytoplasmic vesicles. Cancer Res 58: 4766–4770. 9809973

[3] Dibble CC, Elis W, Menon S, Qin W, Klekota J, et al (2012) TBC1D7 is a third subunit of the TSC1-TSC2 complex upstream of mTORC1. Mol Cell 47: 535–546. 22795129 10.1016/j.molcel.2012.06.009 PMC3693578

[4] Duvel K, Yecies JL, Menon S, Raman P, Lipovsky AI, et al (2010) Activation of a metabolic gene regulatory network downstream of mTOR complex 1. Mol Cell 39: 171–183. 20670887 10.1016/j.molcel.2010.06.022 PMC2946786

[5] Duffy K, Al-Saleem T, Karbowniczek M, Ewalt D, Prowse AH, et al (2002) Mutational analysis of the von hippel lindau gene in clear cell renal carcinomas from tuberous sclerosis complex patients. Mod Pathol 15: 205–210. 11904337 10.1038/modpathol.3880517

[6] El-Hashemite N, Walker V, Zhang H, Kwiatkowski DJ (2003) Loss of Tsc1 or Tsc2 induces vascular endothelial growth factor production through mammalian target of rapamycin. Cancer Res 63: 5173–5177. 14500340

[7] Karbowniczek M, Zitserman D, Khabibullin D, Hartman T, Yu J, et al (2010) The evolutionarily conserved TSC/Rheb pathway activates Notch in tuberous sclerosis complex and Drosophila external sensory organ development. J Clin Invest 120: 93–102. 20038815 10.1172/JCI40221 PMC2798691

[8] Kenerson HL, Aicher LD, True LD, Yeung RS (2002) Activated mammalian target of rapamycin pathway in the pathogenesis of tuberous sclerosis complex renal tumors. Cancer Res 62: 5645–5650. 12384518

[9] Lee L, Sudentas P, Donohue B, Asrican K, Worku A, et al (2005) Efficacy of a rapamycin analog (CCI-779) and IFN-gamma in tuberous sclerosis mouse models. Genes Chromosomes Cancer 42: 213–227. 15578690 10.1002/gcc.20118

[10] Li C, Lee PS, Sun Y, Gu X, Zhang E, et al (2014) Estradiol and mTORC2 cooperate to enhance prostaglandin biosynthesis and tumorigenesis in TSC2-deficient LAM cells. J Exp Med 211: 15–28. 24395886 10.1084/jem.20131080 PMC3892971

[11] Yu JJ, Robb VA, Morrison TA, Ariazi EA, Karbowniczek M, et al (2009) Estrogen promotes the survival and pulmonary metastasis of tuberin-null cells. Proc Natl Acad Sci U S A 106: 2635–2640. 19202070 10.1073/pnas.0810790106 PMC2637277

[12] Bissler JJ, Kingswood JC, Radzikowska E, Zonnenberg BA, Frost M, et al (2013) Everolimus for angiomyolipoma associated with tuberous sclerosis complex or sporadic lymphangioleiomyomatosis (EXIST-2): a multicentre, randomised, double-blind, placebo-controlled trial. Lancet 381: 817–824. 23312829 10.1016/S0140-6736(12)61767-X

[13] Bissler JJ, McCormack FX, Young LR, Elwing JM, Chuck G, et al (2008) Sirolimus for angiomyolipoma in tuberous sclerosis complex or lymphangioleiomyomatosis. N Engl J Med 358: 140–151. 18184959 10.1056/NEJMoa063564 PMC3398441

[14] Franz DN, Leonard J, Tudor C, Chuck G, Care M, et al (2006) Rapamycin causes regression of astrocytomas in tuberous sclerosis complex. Ann Neurol 59: 490–498. 16453317 10.1002/ana.20784

[15] McCormack FX, Inoue Y, Moss J, Singer LG, Strange C, et al (2011) Efficacy and safety of sirolimus in lymphangioleiomyomatosis. N Engl J Med 364: 1595–1606. 21410393 10.1056/NEJMoa1100391 PMC3118601

[16] Dalle Pezze P, Sonntag AG, Thien A, Prentzell MT, Godel M, et al (2012) A dynamic network model of mTOR signaling reveals TSC-independent mTORC2 regulation. Sci Signal 5: ra25. 22457331 10.1126/scisignal.2002469

[17] Karbowniczek M, Cash T, Cheung M, Robertson GP, Astrinidis A, et al (2004) Regulation of B-Raf kinase activity by tuberin and Rheb is mammalian target of rapamycin (mTOR)-independent. J Biol Chem 279: 29930–29937. 15150271 10.1074/jbc.M402591200

[18] Karbowniczek M, Robertson GP, Henske EP (2006) Rheb inhibits C-raf activity and B-raf/C-raf heterodimerization. J Biol Chem 281: 25447–25456. 16803888 10.1074/jbc.M605273200

[19] Hartman TR, Liu D, Zilfou JT, Robb V, Morrison T, et al (2009) The tuberous sclerosis proteins regulate formation of the primary cilium via a rapamycin-insensitive and polycystin 1-independent pathway. Hum Mol Genet 18: 151–163. 18845692 10.1093/hmg/ddn325 PMC2644647

[20] Akbaraly TN, Hamer M, Ferrie JE, Lowe G, Batty GD, et al (2013) Chronic inflammation as a determinant of future aging phenotypes. CMAJ 185: E763–770. 24043651 10.1503/cmaj.122072 PMC3826354

[21] Liu Y, Marks K, Cowley GS, Carretero J, Liu Q, et al (2013) Metabolic and Functional Genomic Studies Identify Deoxythymidylate Kinase as a Target in LKB1-Mutant Lung Cancer. Cancer Discov 3: 870–879. 23715154 10.1158/2159-8290.CD-13-0015 PMC3753578

[22] FitzGerald GA, Patrono C (2001) The coxibs, selective inhibitors of cyclooxygenase-2. N Engl J Med 345: 433–442. 11496855 10.1056/NEJM200108093450607

[23] Pacheco-Rodriguez G, Steagall WK, Crooks DM, Stevens LA, Hashimoto H, et al (2007) TSC2 loss in lymphangioleiomyomatosis cells correlated with expression of CD44v6, a molecular determinant of metastasis. Cancer Res 67: 10573–10581. 17975002 10.1158/0008-5472.CAN-07-1356 PMC10068840

[24] Muller R (2004) Crosstalk of oncogenic and prostanoid signaling pathways. J Cancer Res Clin Oncol 130: 429–444. 15205946 10.1007/s00432-004-0570-y PMC12161876

[25] Landi MT, Dracheva T, Rotunno M, Figueroa JD, Liu H, et al (2008) Gene expression signature of cigarette smoking and its role in lung adenocarcinoma development and survival. PLoS One 3: e1651. 18297132 10.1371/journal.pone.0001651 PMC2249927

[26] Barnes EA, Kenerson HL, Jiang X, Yeung RS (2010) Tuberin regulates E-cadherin localization: implications in epithelial-mesenchymal transition. Am J Pathol 177: 1765–1778. 20813961 10.2353/ajpath.2010.090233 PMC2947273

[27] Pacheco-Rodriguez G, Kumaki F, Steagall WK, Zhang Y, Ikeda Y, et al (2009) Chemokine-enhanced chemotaxis of lymphangioleiomyomatosis cells with mutations in the tumor suppressor TSC2 gene. J Immunol 182: 1270–1277. 19155472 10.4049/jimmunol.182.3.1270 PMC2947111

[28] Zhang H, Cicchetti G, Onda H, Koon HB, Asrican K, et al (2003) Loss of Tsc1/Tsc2 activates mTOR and disrupts PI3K-Akt signaling through downregulation of PDGFR. J Clin Invest 112: 1223–1233. 14561707 10.1172/JCI17222 PMC213485

[29] Yu J, Astrinidis A, Howard S, Henske EP (2004) Estradiol and tamoxifen stimulate LAM-associated angiomyolipoma cell growth and activate both genomic and nongenomic signaling pathways. Am J Physiol Lung Cell Mol Physiol 286: L694–700. 12922981 10.1152/ajplung.00204.2003

[30] Lee PS, Tsang SW, Moses MA, Trayes-Gibson Z, Hsiao LL, et al (2010) Rapamycin-insensitive up-regulation of MMP2 and other genes in tuberous sclerosis complex 2-deficient lymphangioleiomyomatosis-like cells. Am J Respir Cell Mol Biol 42: 227–234. 19395678 10.1165/rcmb.2009-0050OC PMC2822984

[31] Everitt JI, Wolf DC, Howe SR, Goldsworthy TL, Walker C (1995) Rodent model of reproductive tract leiomyomata. Clinical and pathological features. Am J Pathol 146: 1556–1567. 7778693 PMC1870902

[32] Howe SR, Gottardis MM, Everitt JI, Walker C (1995) Estrogen stimulation and tamoxifen inhibition of leiomyoma cell growth in vitro and in vivo. Endocrinology 136: 4996–5003. 7588234 10.1210/endo.136.11.7588234

[33] Onda H, Lueck A, Marks PW, Warren HB, Kwiatkowski DJ (1999) Tsc2(+/−) mice develop tumors in multiple sites that express gelsolin and are influenced by genetic background. J Clin Invest 104: 687–695. 10491404 10.1172/JCI7319 PMC408440

[34] Lu TP, Tsai MH, Lee JM, Hsu CP, Chen PC, et al (2010) Identification of a novel biomarker, SEMA5A, for non-small cell lung carcinoma in nonsmoking women. Cancer Epidemiol Biomarkers Prev 19: 2590–2597. 20802022 10.1158/1055-9965.EPI-10-0332

[35] Young L, Lee HS, Inoue Y, Moss J, Singer LG, et al (2013) Serum VEGF-D a concentration as a biomarker of lymphangioleiomyomatosis severity and treatment response: a prospective analysis of the Multicenter International Lymphangioleiomyomatosis Efficacy of Sirolimus (MILES) trial. Lancet Respir Med 1: 445–452. 24159565 10.1016/S2213-2600(13)70090-0 PMC3804556

[36] Young LR, Vandyke R, Gulleman PM, Inoue Y, Brown KK, et al (2010) Serum vascular endothelial growth factor-D prospectively distinguishes lymphangioleiomyomatosis from other diseases. Chest 138: 674–681. 20382711 10.1378/chest.10-0573 PMC2940071

[37] Howe SR, Gottardis MM, Everitt JI, Goldsworthy TL, Wolf DC, et al (1995) Rodent model of reproductive tract leiomyomata. Establishment and characterization of tumor-derived cell lines. Am J Pathol 146: 1568–1579. 7539981 PMC1870894

[38] Astrinidis A, Cash TP, Hunter DS, Walker CL, Chernoff J, et al (2002) Tuberin, the tuberous sclerosis complex 2 tumor suppressor gene product, regulates Rho activation, cell adhesion and migration. Oncogene 21: 8470–8476. 12466966 10.1038/sj.onc.1205962

[39] Kang SA, Pacold ME, Cervantes CL, Lim D, Lou HJ, et al (2013) mTORC1 phosphorylation sites encode their sensitivity to starvation and rapamycin. Science 341: 1236566. 23888043 10.1126/science.1236566 PMC3771538

[40] Thoreen CC, Kang SA, Chang JW, Liu Q, Zhang J, et al (2009) An ATP-competitive mammalian target of rapamycin inhibitor reveals rapamycin-resistant functions of mTORC1. J Biol Chem 284: 8023–8032. 19150980 10.1074/jbc.M900301200 PMC2658096

[41] Huang Z, Laliberte F, Tremblay NM, Weech PK, Street IP (1994) A continuous fluorescence-based assay for the human high-molecular-weight cytosolic phospholipase A2. Anal Biochem 222: 110–115. 7856835 10.1006/abio.1994.1461

[42] Yuan C, Rieke CJ, Rimon G, Wingerd BA, Smith WL (2006) Partnering between monomers of cyclooxygenase-2 homodimers. Proc Natl Acad Sci U S A 103: 6142–6147. 16606823 10.1073/pnas.0601805103 PMC1458845

[43] Burke JE, Dennis EA (2009) Phospholipase A2 structure/function, mechanism, and signaling. J Lipid Res 50 Suppl: S237–242. 19011112 10.1194/jlr.R800033-JLR200 PMC2674709

[44] Burke JE, Dennis EA (2009) Phospholipase A2 biochemistry. Cardiovasc Drugs Ther 23: 49–59. 18931897 10.1007/s10557-008-6132-9 PMC2823292

[45] Prevost N, Mitsios JV, Kato H, Burke JE, Dennis EA, et al (2009) Group IVA cytosolic phospholipase A2 (cPLA2alpha) and integrin alphaIIbbeta3 reinforce each other’s functions during alphaIIbbeta3 signaling in platelets. Blood 113: 447–457. 18840708 10.1182/blood-2008-06-162032 PMC2615656

[46] Duncan RE, Sarkadi-Nagy E, Jaworski K, Ahmadian M, Sul HS (2008) Identification and functional characterization of adipose-specific phospholipase A2 (AdPLA). J Biol Chem 283: 25428–25436. 18614531 10.1074/jbc.M804146200 PMC2533091

[47] Jaworski K, Ahmadian M, Duncan RE, Sarkadi-Nagy E, Varady KA, et al (2009) AdPLA ablation increases lipolysis and prevents obesity induced by high-fat feeding or leptin deficiency. Nat Med 15: 159–168. 19136964 10.1038/nm.1904 PMC2863116

[48] Krymskaya VP (2008) Smooth muscle-like cells in pulmonary lymphangioleiomyomatosis. Proc Am Thorac Soc 5: 119–126. 18094094 10.1513/pats.200705-061VS PMC2645298

[49] Zhe X, Schuger L (2004) Combined smooth muscle and melanocytic differentiation in lymphangioleiomyomatosis. J Histochem Cytochem 52: 1537–1542. 15557209 10.1369/jhc.4A6438.2004

[50] Matsumoto Y, Horiba K, Usuki J, Chu SC, Ferrans VJ, et al (1999) Markers of cell proliferation and expression of melanosomal antigen in lymphangioleiomyomatosis. Am J Respir Cell Mol Biol 21: 327–336. 10460750 10.1165/ajrcmb.21.3.3693

[51] Goncharova EA, Goncharov DA, Lim PN, Noonan D, Krymskaya VP (2006) Modulation of cell migration and invasiveness by tumor suppressor TSC2 in lymphangioleiomyomatosis. Am J Respir Cell Mol Biol 34: 473–480. 16388022 10.1165/rcmb.2005-0374OC PMC2644208

[52] Karbowniczek M, Yu J, Henske EP (2003) Renal angiomyolipomas from patients with sporadic lymphangiomyomatosis contain both neoplastic and non-neoplastic vascular structures. Am J Pathol 162: 491–500. 12547707 10.1016/S0002-9440(10)63843-6 PMC1851167

[53] Neuman NA, Henske EP (2011) Non-canonical functions of the tuberous sclerosis complex-Rheb signalling axis. EMBO Mol Med 3: 189–200. 21412983 10.1002/emmm.201100131 PMC3377068

[54] Huang J, Dibble CC, Matsuzaki M, Manning BD (2008) The TSC1-TSC2 complex is required for proper activation of mTOR complex 2. Mol Cell Biol 28: 4104–4115. 18411301 10.1128/MCB.00289-08 PMC2423120

